# Expression of thrombospondin-4 in the infrapatellar fat pad and synovial fluid – potential contribution to osteoarthritis pain

**DOI:** 10.1186/s13075-025-03615-7

**Published:** 2025-08-26

**Authors:** Sebastian Braun, Patrizia Pollinger, Rebecca Sohn, Anna E. Rapp, Gundula Rösch, Frank Zaucke, Zsuzsa Jenei-Lanzl

**Affiliations:** 1 Dr. Rolf M. Schwiete Research Unit for Osteoarthritis, Department of Trauma Surgery and Orthopedics, Goethe University Frankfurt, University Hospital, Marienburgstraße 2, D-60528 Frankfurt am Main, Germany; 2 Center for Musculoskeletal Surgery, Charité – Universitätsmedizin Berlin, Corporate Member of Freie Universität Berlin and Humboldt-Universität zu Berlin, Charitéplatz 1, 10117 Berlin, Germany

**Keywords:** Fibrosis, Infrapatellar fat pad, Osteoarthritis, Pain, Synovial fluid, Thrombospondin-4

## Abstract

**Background:**

During osteoarthritis (OA) pathogenesis, the infrapatellar fat pad (IFP) undergoes fibrotic changes that might contribute to pain development. Recent studies have demonstrated that thrombospondin-4 (TSP-4), first detected in the extracellular matrix of cartilage and released during its degradation, has been implicated in the pathogenesis of pain. Therefore, we analyzed TSP-4 levels in the IFP and synovial fluid and correlated this data with IFP fibrosis and knee joint pain.

**Methods:**

IFP and synovial fluid were collected from patients undergoing total knee replacement surgery. Total WOMAC total and pain scores were determined preoperatively. IFP sections were stained using standard Masson trichrome and hematoxylin/eosin dyes to assess fibrotic changes, number of vessels and lymphocytic infiltration. TSP-4 expression in the IFP was detected immunohistochemically. TSP-4 in synovial fluid samples was quantified using ELISA.

**Results:**

TSP-4 was detectable in human IFP tissue at the protein level and its expression levels showed a positive correlation with the degree of tissue fibrosis. Regarding the degree of fibrosis and TSP-4-stained areas, four patient subgroups could be distinguished. Notably, moderate levels of TSP-4 expression were already detectable in samples exhibiting a low degree of fibrosis. There was no significant correlation between TSP-4 staining intensity in IFP and pain. There was no correlation between TSP-4 staining intensity and synovial fluid TSP-4 concentrations. A significant relationship between synovial fluid TSP-4 concentration and pain intensity was only found in female OA patients.

**Conclusions:**

TSP-4 has been detected in the IFP for the first time. The correlation between TSP-4 expression and fibrotic severity indicates a possible involvement of TSP-4 in the development of fibrosis. Although TSP-4 within the IFP may not directly mediate pain, its presence in synovial fluid could be of functional relevance in pain-related mechanisms. Further analysis of synovial fluid and even serum samples from larger patient populations will determine whether TSP-4 could serve as a biomarker for pain or potentially represent a novel target for analgesic therapies.

**Supplementary Information:**

The online version contains supplementary material available at 10.1186/s13075-025-03615-7.

## Background

Osteoarthritis (OA) is the most common chronic degenerative joint disease characterized by limited joint function and chronic joint pain, the cardinal symptom of OA [1-3]. Systemic determinants such as age, sex, genetic factors, metabolic disorders as well as local influences [2] like joint overloading by heavy physical or excessive work and overweight are well known risk factors leading to OA [4]. Current symptomatic therapeutic options include long-term analgesics treatment or, in many cases, total joint replacement surgery as final intervention [5]. Since no causal therapy for OA is available at present, the knowledge of OA risk factors as well as pathomechanisms has to be improved. During OA pathogenesis degenerative and fibrotic changes occur not only in articular cartilage, but also in other joint tissues such as menisci, synovial tissue, subchondral bone, tendons, ligaments as well as in the infrapatellar fat pad (IFP). However, the exact contribution of the different joint tissue types to disease progression is not yet fully understood.

Current research suggests that the IFP plays a crucial role in the pathogenesis of OA due its extensive interactions with other intra-articular tissues [2, 6]. In immunohistochemical studies of IFP tissue obtained from knee OA patients, increased fibrosis, hypervascularization, increased lymphocyte infiltration was observed compared to healthy IFP [7]. Besides this, the IFP itself is considered to contribute to OA pathogenesis due to its secretory profile [2]. A degenerative and inflamed IFP produces and releases numerous bioactive molecules such as adipokines [8] impacting disease progression and pain [2]. Besides the well-documented alterations in adipokine release during the progression of OA, significant extracellular matrix changes are evident, particularly with regard to fibrotic transformation [2]. Such fibrotic alterations are predominantly characterized by a marked increase in collagens, reinforcing the fibrous nature of the tissue [9, 10]. Recent studies highlighted the presence and increased expression of non-collageneous matrix proteins such as cartilage oligomeric matrix protein (COMP) also referred to as thrombospondin-5, which is predominantly found in the cartilage matrix and plays a crucial role in the synthesis and remodeling of the extracellular matrix [2, 11]. The fact that COMP substantially contributes to fibrillogenesis of collagen types I and II underlines its potential role in the fibrotic processes occurring during OA [12, 13]. Interestingly, the association of COMP expression with IFP fibrosis suggests a potentially similar role for other members of the thrombospondin family [14].

Thrombospondin-4 (TSP-4) has been shown to bind to various collagen types in articular cartilage [10, 15] and the upregulation of TSP-4 protein in human articular cartilage of OA patients is associated with OA severity [16, 17]. The function of TSP-4 in the cartilage extracellular matrix is not yet fully understood. Several studies suggest that TSP-4 might contribute to the genesis of OA pain [16, 18]. In this regard, previous studies demonstrated that TSP-4 upregulation in response to nerve injuries contributes to neuropathic pain development, and its inhibition can reduce pain [19, 20]. Furthermore, studies confirmed a role of TSP-4 in orofacial and joint-mediated pain, and its impact on spinal cord synaptogenesis emphasizes its importance in OA-related neuropathic pain [20-24]. Until now, no studies investigated the expression or function of TSP-4 in the IFP.

We hypothesize that, similarly to COMP, also TSP-4 is detectable in the human IFP and its presence might correlate with IFP fibrosis severity and released TSP-4 molecules might induce receptors on sensory neurons in the IFP or in the synovial membrane contributing to the genesis of knee pain in OA patients. Therefore, we analyzed TSP-4 expression in IFP tissue as well as in synovial fluid of patients with late OA and expression levels were correlated with grade of fibrosis and knee pain levels. These results will help to understand the role of TSP-4 in the knee joint and might lead to novel therapeutic strategies for OA by targeting the underlying causes of pain [25, 26].

## Materials and methods

### Patients

Human IFP (*n* = 20) and synovial fluid (*n* = 24) samples were collected from pseudonymized patients undergoing total knee arthroplasty (TKA) at the Trauma Surgery and Orthopedics, Goethe University Frankfurt, University Hospital, Germany. The study population included individuals between 33 and 82 years of age. Exclusion criteria comprised lower limb injuries, any lower limb surgeries within 6 months prior to study participation, former endoprosthetic surgeries of the hip or ankle joint, rheumatoid arthritis, or various neurological disorders such as Alzheimer’s disease, cerebral palsy, epilepsy, Huntington’s disease, multiple sclerosis, muscular dystrophy, paraneoplastic syndrome, Parkinson’s disease, poliomyelitis, stroke with paralysis, and tremor. During TKA, the entire IFP was resected in a standardized manner. The specimen was marked with sutures to preserve anatomical orientation (proximal, distal, medial, lateral, and articular surface). For histological analysis, tissue samples approximately 10 × 5 × 5 mm in size were obtained from five anatomically distinct regions of the IFP to account for its known heterogeneity. Sampling was performed in a consistent and standardized fashion to ensure reproducibility and comparability across patients. In addition, the medical chart of each patient was reviewed for information regarding age, sex, and current pain medications. The pain level of OA patients was assessed preoperatively using the Western Ontario and McMaster Universities Osteoarthritis Index (WOMAC). The patients were selected so that half of them had lower and the other half had higher WOMAC pain scores. The basic patient information is summarized in Tables 1 and 2 for IFP and synovial fluid samples respectively (IFP and synovial fluid partly from the same patients). All experiments were conducted in accordance with the Declaration of Helsinki, and approved by the Ethics Committee of the Department of Medicine of the Goethe University Frankfurt (vote Nr.: 19–347).

**Table 1:** IFP Samples - Patient characteristics and analgesic medication

Patient Characteristics	
Patients, n	20
Sex, no. female (%) / no. male (%)	10 (50%) / 10 (50%)
Age [years], mean (± SD, range)	64.2 (± 13.4, 33–82)
BMI [kg/m^2^], mean (± SD, range)	31.8 (± 7.3, 19.6–44)
WOMAC total	126 (± 65, 50–223)
WOMAC pain	5.8 (± 3.5, 1.6–8.9)
Painkiller: NSAID/opioid, n (%)	7 (35%)

**Table 2:** Synovial fluid samples - Patient characteristics and analgesic medication

Patient Characteristics	
Patients, n	24
Sex, no. female (%) / no. male (%)	12 (50%) / 12 (50%)
Age [years], mean (± SD, range)	63.9 (± 10.4, 39–80)
BMI [kg/m^2^], mean (± SD, range)	33.5 (± 7.2, 20–48)
WOMAC total	138 (± 61, 52–237)
WOMAC pain	6.1 (± 4.2, 2.9–10.2)
Pankiller: NSAID/opioid, n (%)	12 (50%)

### Tissue processing

IFP samples were fixed in 4% paraformaldehyde (Merck, Darmstadt, Hessen, Germany) for 24 h, washed in 1x PBS (Sigma-Aldrich, St. Louis, USA) overnight on a shaker and embedded in paraffin (Paraplast PLUS, Merck, Darmstadt, Hessen, Germany). Sections of 8 μm were then deparaffinized using a standard protocol with xylene (Sigma-Aldrich, St. Louis, USA), gradually rehydrated through alcohol series and stained as described below.

Synovial fluid samples of twelve female and twelve male were frozen immediately after knee joint punction in 100–500 µl aliquots and stored at -80 °C until analysis.

### Histology and immunohistochemistry

Deparaffinized IFP sections were stained with standard hematoxylin and eosin (HE) and Masson trichrome to assess structural and fibrotic changes. For immunohistochemistry, sections were blocked using 2.5% normal horse serum (Vector Laboratories, Burlingame, USA) for 20 min at room temperature. The sections were then incubated overnight at 4 C with a primary antibody in 1:500 dilution against TSP-4 (rabbit anti-rat antibody raised against recombinantly expressed full-length TSP-4, diluted 1:500 in 1% bovine serum albumin (BSA)). Negative control stainings without addition of the primary antibody were carried out to exclude unspecific binding of the secondary antibody (supplementary Fig. 1). Detection was performed using an HRP-conjugated secondary antibody in 1:2000 dilution (Goat anti-rabbit IgG HRP-linked #7074, Cell Signaling Technology, Danvers, USA) and visualized with DAB (3,3’-Diaminobenzidine, Sigma-Aldrich) staining kit.

### Quantitative analysis of fibrotic changes and TSP-4 expression

**Fig. 1:**
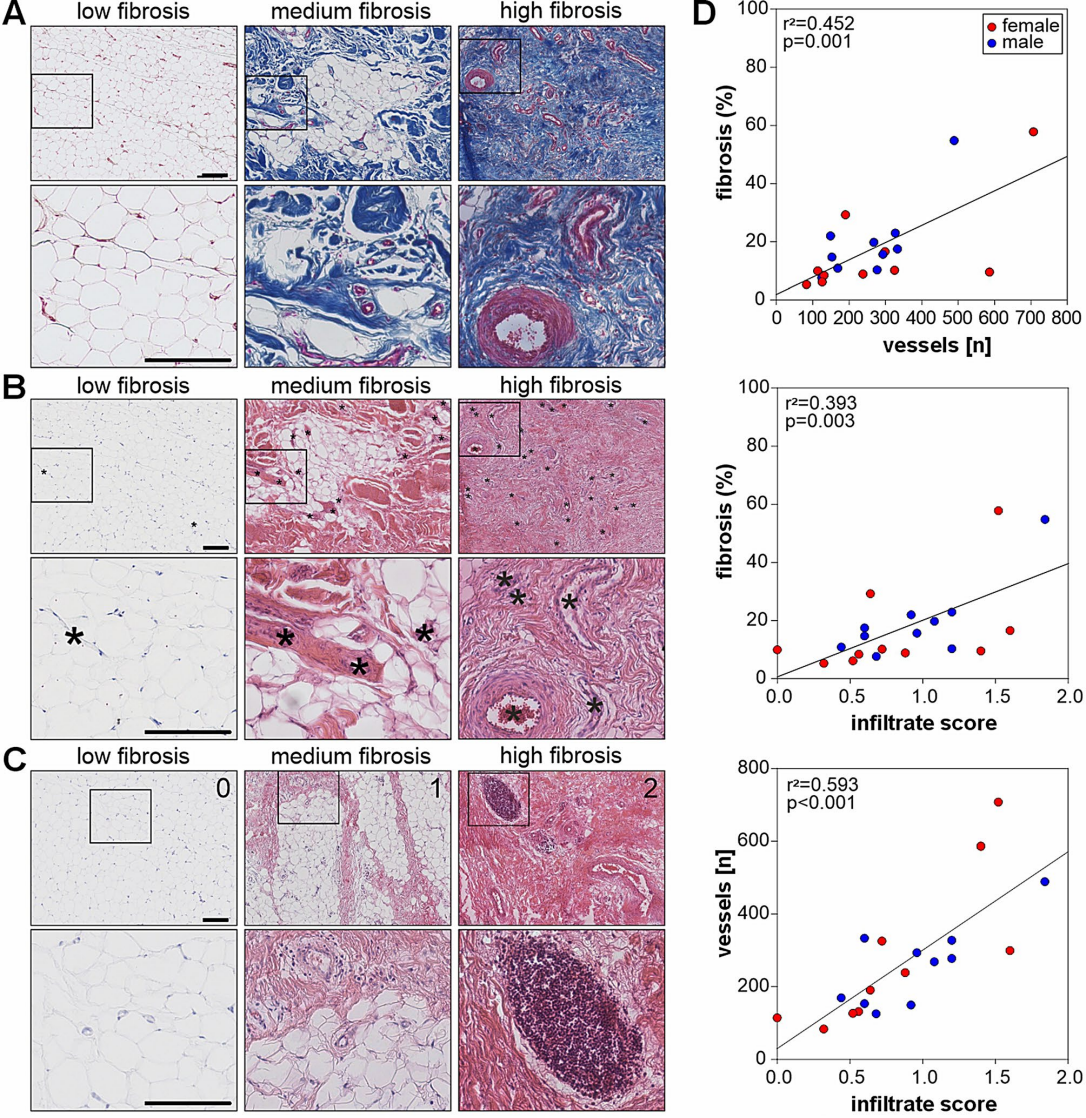
Histological analyses of IFP samples. Representative images and magnifications of (**A**) Masson trichome-stained sections from three different patients to analyze the area of fibrosis (blue) as well as HE-stained sections to (**B**) quantify the number of vessels and to (**C**) score the infiltrate according to [7] with 0, no infiltrate; 1, perivascular infiltrate only; 2, perivascular and interstitial infiltrate; (scale bar in all panels 200 μm). (**D**) Correlations between the percentage of fibrosis, vessel number as well as lymphocytic infiltrate score (*n* = 20). Each circle corresponds to the mean value for an individual patient with red circles indicating female patients and blue circles male patients. r^2^ values indicate the coefficient of determination and p values indicate the level of significance

Tissue sections stained with HE and Masson trichome were used to quantify the grade of fibrosis and analyzed using a microscope (Nikon Eclipse Ti, Nikon, Tokyo, Japan). IFP areas positively stained for TSP-4 were quantified. Five randomly selected sections per IFP sample and patient with a minimal distance of 150 μm between two sections and five randomly chosen fields of view per each section were analyzed. Thus, 25 images per tissue sample were evaluated for each patient. Blue fibrotic and brown TSP-4-positive areas were quantified using Fiji (ImageJ) software (version 1.52p). Positively stained areas were expressed as a percentage of the total area of each picture. Then, the mean value of 25 images was calculated for each patient. In order to quantify vascularization, the blood vessels were counted in 25 Masson trichrome stained images and the sum was calculated for each patient. In addition, the presence of a lymphatic infiltrate was examined in HE-stained sections by following scoring [7] 0: no infiltrate, 1: infiltrate only perivascular, and 2: infiltrate perivascular and interstitial (Fig. 1). Mean values were calculated for all the 25 sections of each patient.

### TSP-4 in synovial fluid

The concentration of TSP-4 in synovial fluid samples diluted 1:100 was quantified in triplicates using a commercially available ELISA kit (Human TSP-4 ELISA Kit #EH473RB, Thermo Fisher, Darmstadt, Germany) according to the manufacturer´s protocol.

### Statistical analysis

Data are presented as box plots or scatter plots with regression lines. To test for normality, the Kolmogorov–Smirnov test was used. Differences between two independent groups were calculated using the Student´s t- test for normally distributed data or Mann-Whitney-U test when normality was not given. Differences between more than two independent groups were calculated using the non-parametric ANOVA (when data were normally distributed) or ANOVA on ranks (when normality was not given) followed by the Bonferroni or Student–Newman–Keuls correction method (pairwise comparison of all groups), as suggested for the respective analysis by the statistics software (SigmaPlot V.11, Systat Software, Erkrath, Germany). Variant and invariant correlations between parameters were determined by assessing the Spearman’s rank correlation coefficient (Rs) and respective p-values were obtained using SigmaPlot. p values less than 0.05 were considered significant.

## Results

### Correlations between fibrotic changes, vascularization, and lymphocytic infiltration

Histological analyses demonstrate a large variability in fibrosis, vascularization and lymphocyte infiltration. All stages of fibrosis could be detected and the number of vessels as well as lymphocyte infiltration increased with grade of fibrosis (Fig. 1A-C). The mean percentage of fibrotic to total tissue area was 17.84% ± 14.55% (range: 5.24–57.77%), mean total vessels count was 269.1 ± 164.92 (range: 83 to 707) and mean lymphocyte infiltration score was 0.88 ± 0.47 (range: 0 to 1.84). In addition, a significant positive correlation between the number of vessels and the percentage of fibrosis (r^2^ = 0.452; *p* = 0.001), between the lymphocytic infiltration score and the percentage of fibrosis (r^2^ = 0.393; *p* = 0.003), and between the lymphocytic infiltration score and the number of vessels (r^2^ = 0.593; *p* < 0.001) was detected (Fig. 1D).

### Relationship between BMI, age, as well as sex and the grade of fibrosis

**Fig. 2:**
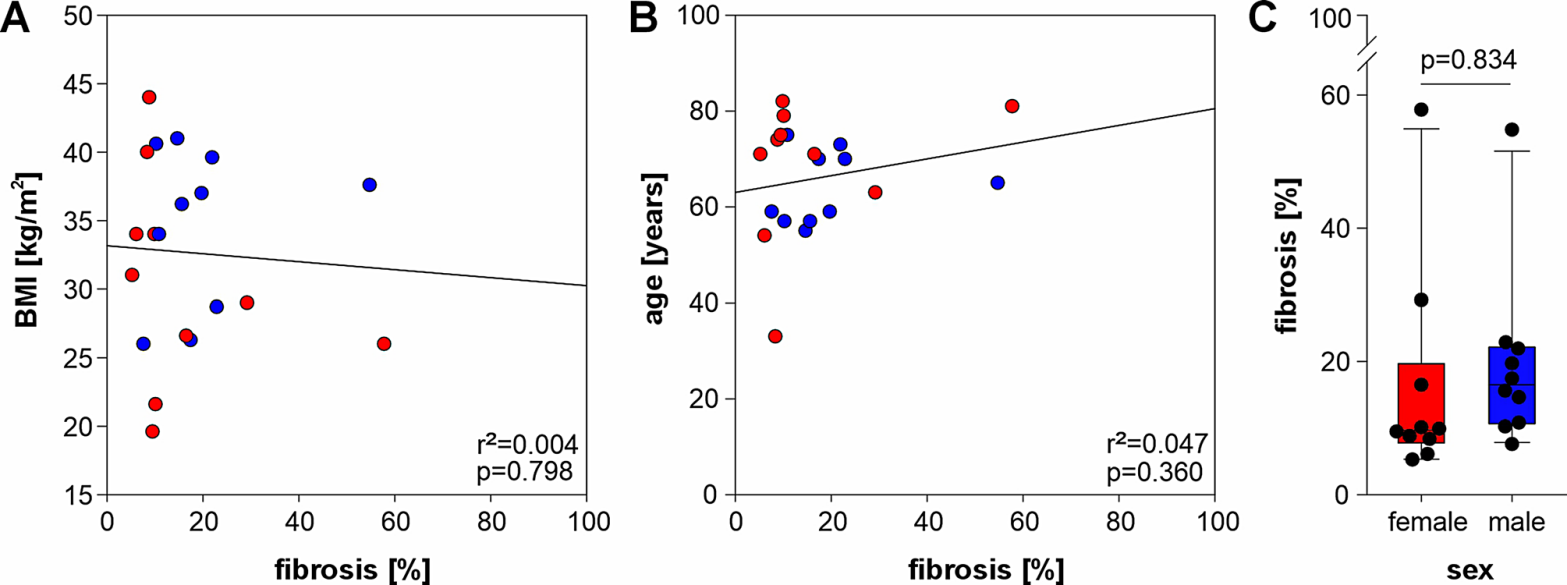
Correlation analyses of IFP fibrosis with patient-specific parameters. Correlation between (**A**) body mass index (BMI) and (**B**) age with the percentage of IFP fibrosis (*n* = 20). Each circle represents an individual patient, with red circles indicating female patients (*n* = 10) and blue circles male patients (*n* = 10). (**C**) Influence of sex on the grade of IFP fibrosis (*n* = 20). Data are presented as box plots with whiskers, where each black circle represents an individual patient. r^2^ values indicate the coefficient of determination and p values indicate the level of significance

Since both the number of vessels and the lymphocytic infiltration score increased with the grade of fibrosis, only the latter was correlated with further parameters in the following. The percentage of fibrosis correlated neither with BMI nor with age (Fig. 2A, B). Furthermore, no significant sex-specific differences in the grade of fibrosis were observed (Fig. 2C).

### Correlation between TSP-4 expression and fibrosis

**Fig. 3:**
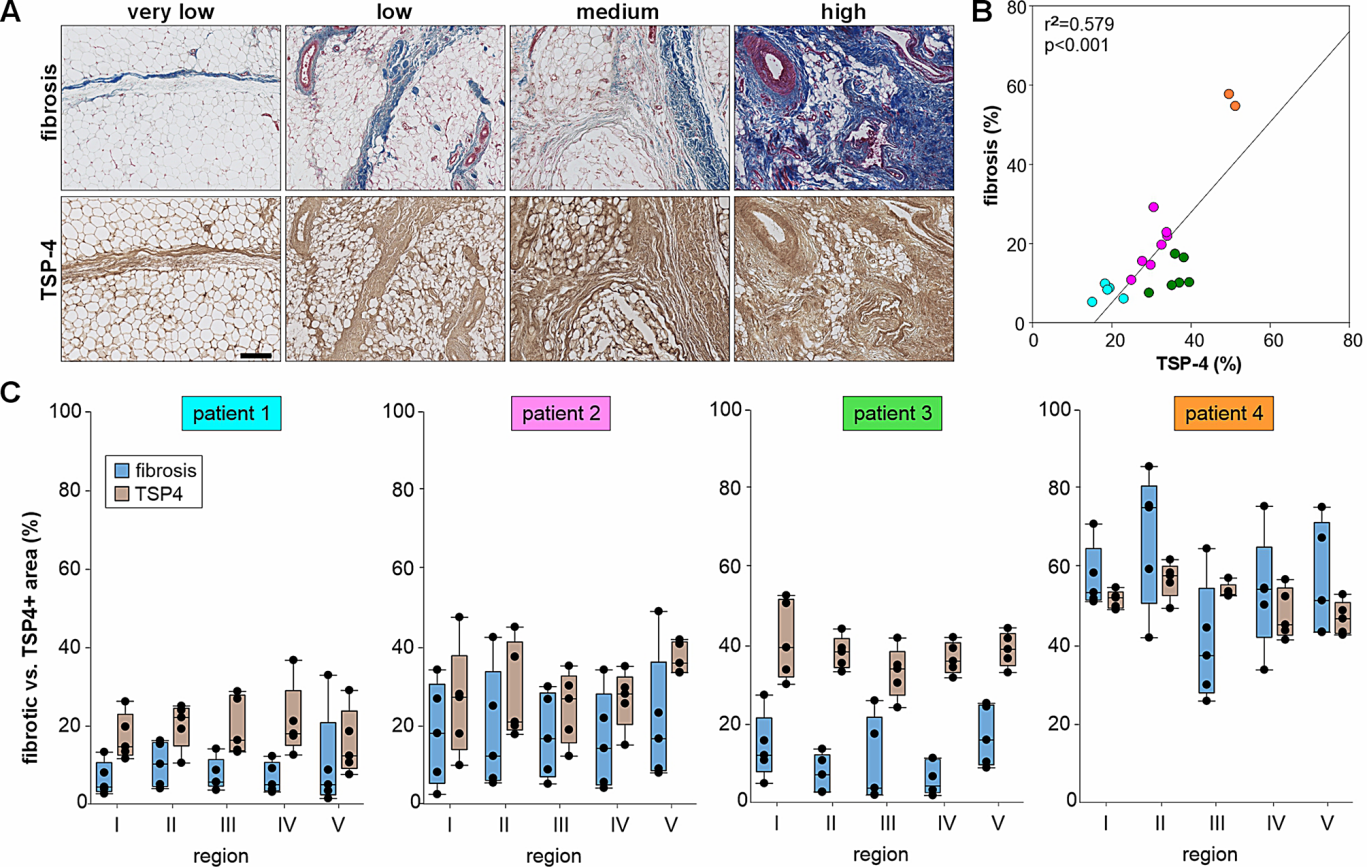
Correlation between grade of fibrosis and TSP-4 expression in the IFP. (**A**) Detection of TSP-4 in IFP samples with different grades of fibrosis (scale bar 200 μm). (**B**) Correlation between the grade of fibrosis and the TSP-4 positively stained area in all patients included in this study (*n* = 20). (**C**) Quantification of fibrosis and TSP-4 staining in selected patients representing the four identified patient groups: light blue/patient 1 – very low fibrosis and low TSP-4 expression; pink/patient 2 - medium levels of fibrosis and medium TSP-4 expression; green/patient 3 - fibrosis but medium TSP-4 expression; orange/patient 4 - very high fibrosis and high TSP-4 expression (*n* = 20). Each circle represents one individual patient, r^2^ indicates the coefficient of determination and p value indicates the level of significance

TSP-4 could be detected in all IFP samples in fibrotic areas. The mean percentage of the IFP tissue area positively stained for TSP-4 was 31.16% ± 9.70% (range: 15.04–51.17%). In general, increased fibrosis was associated with increased TSP-4 expression (Fig. 3A). The fact that the intensity of the TSP-4 staining does not decrease in strongly enlarged fibrotic areas suggests that the synthesis of TSP-4 is upregulated. Even though there was a significant positive correlation between grade of fibrosis and TSP-4-positive area (Fig. 3B), four distinct groups of patients could be identified regarding the grade of fibrosis and TSP-4-positive area: I - very low fibrosis (0–10%) and low TSP-4 values, II - low to medium fibrosis (10–30%) and low TSP-4 values, III – low to medium medium fibrosis (10–30%) and medium TSP-4 values, IV – high fibrosis (> 40%) and high TSP-4 values (Fig. 3C).

### Correlation between pain and fibrosis or TSP-4 expression

**Fig. 4:**
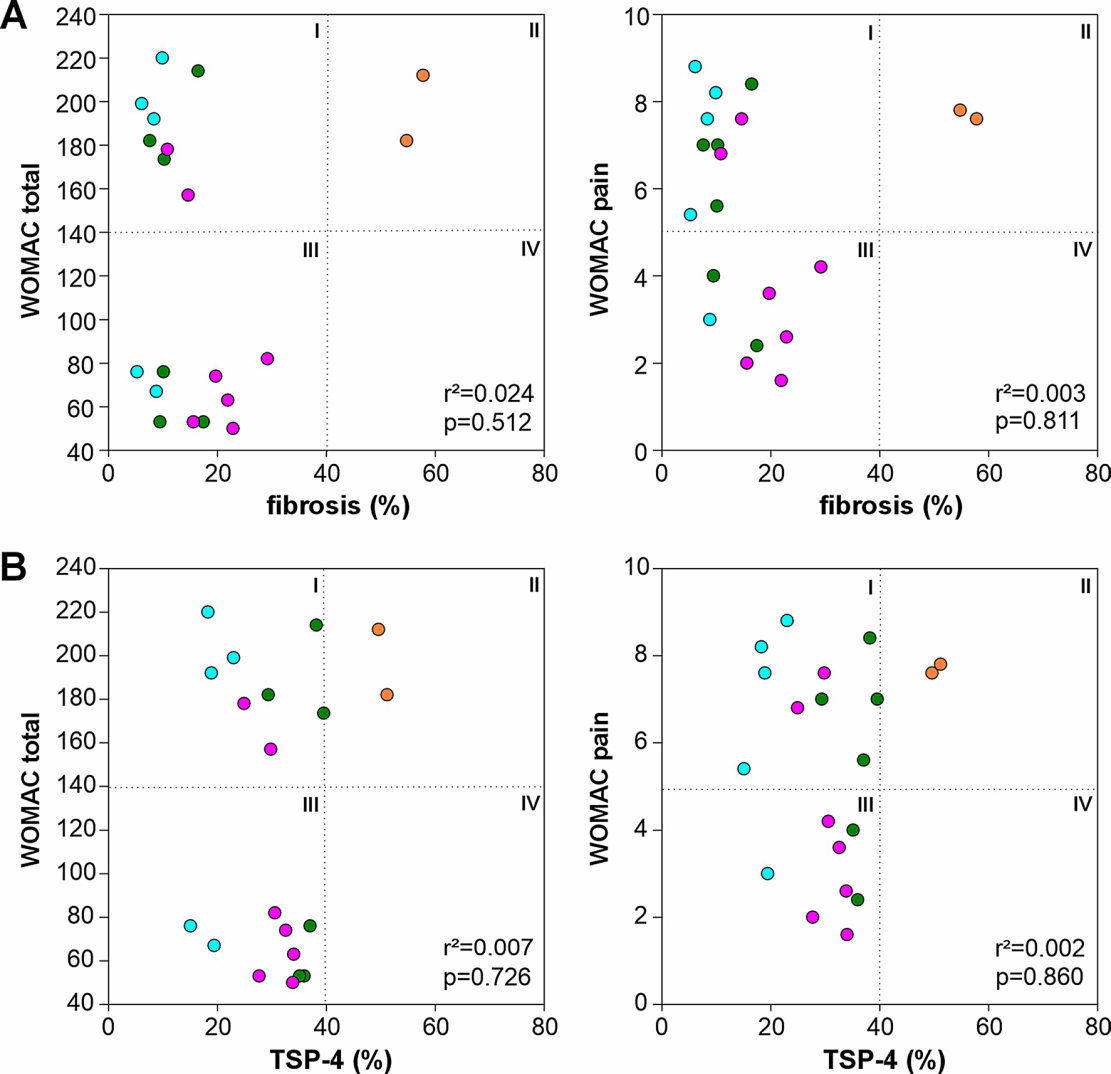
Correlation between WOMAC scores and fibrosis or TSP-4 expression in the IFP. (**A**) Correlation between the grade of fibrosis and the total WOMAC and WOMAC pain scores (*n* = 20). (**B**) Correlation between TSP-4 expression levels and total WOMAC and WOMAC pain scores (*n* = 20). Each circle represents an individual patient, categorized into four groups based on their histopathological and clinical severity scores as described in the legend to Fig. 3: light blue/patient 1 – very low fibrosis and low TSP-4 expression; pink/patient 2 - medium levels of fibrosis and medium TSP-4 expression; green/patient 3 - fibrosis but medium TSP-4 expression; orange/patient 4 - very high fibrosis and high TSP-4 expression. r^2^ values indicate the coefficient of determination and p values indicate the level of significance

There was no significant association between the grade of fibrosis and either the total WOMAC score or WOMAC pain score. The scatter plots were segmented into four quadrants indicating low or high total WOMAC and WOMAC pain scores in relation to low or high grade of fibrosis and TSP-4 expression, respectively. This closer examination of data distribution across the quadrants revealed an absence of points in quadrant IV and a sparse presence in quadrant II for both parameters. This pattern might suggest that a higher degree of fibrosis (> 40%) does not coincide with lower total WOMAC or WOMAC pain scores (Fig. 4A). A similar trend was observed with TSP-4: there were no patients with high TSP-4 expression paired with low pain or low total WOMAC scores (Fig. 4B).

### Relationship between BMI, age, as well as sex and the concentration of TSP-4 in the synovial fluid

**Fig. 5:**
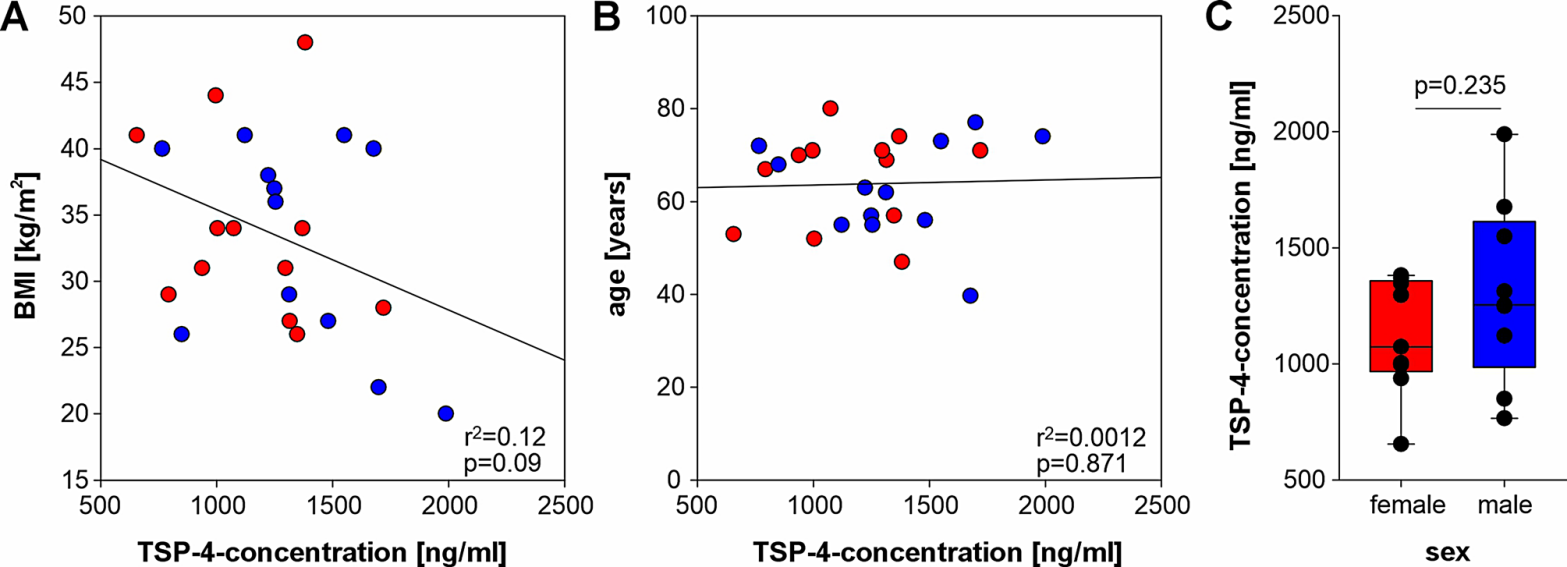
Correlation analyses of synovial fluid TSP-4 levels with patient-specific parameters. Correlation between (**A**) body mass index (BMI) and (**B**) age with the concentration of TSP-4 in the synovial fluid (*n* = 24). Each circle represents an individual patient, with red circles indicating female patients (*n* = 12) and blue circles male patients (*n* = 12). (**C**) Influence of sex on the concentration of TSP-4 in the synovial fluid (*n* = 24). Data are presented as box plots with whiskers, where each black circle represents an individual patient. r^2^ values indicate the coefficient of determination and p values indicate the level of significance

Before analyzing the relationship of synovial fluid TSP-4 levels and pain scores, the potential influence of basic clinical data was investigated. The concentration of TSP-4 did not correlate with basic clinicalparameters such as BMI and age (Fig. 5A, B). Similarly, no significant sex-specific differences in the synovial fluid TSP-4 levels were observed (Fig. 5C).

### Correlation between TSP-4 concentrations in synovial fluid and WOMAC scores

TSP-4 could be detected in synovial fluid samples using ELISA. The mean TSP-4 concentration was 1,252.17 ± 334.45 ng/ml, ranging from 655 to 1,988 ng/ml. TSP-4 concentration in female patients correlated significantly and positively with the total WOMAC and WOMAC pain scores. In contrast, male patients did not exhibit such a correlation (Fig. 5).

**Fig. 6:**
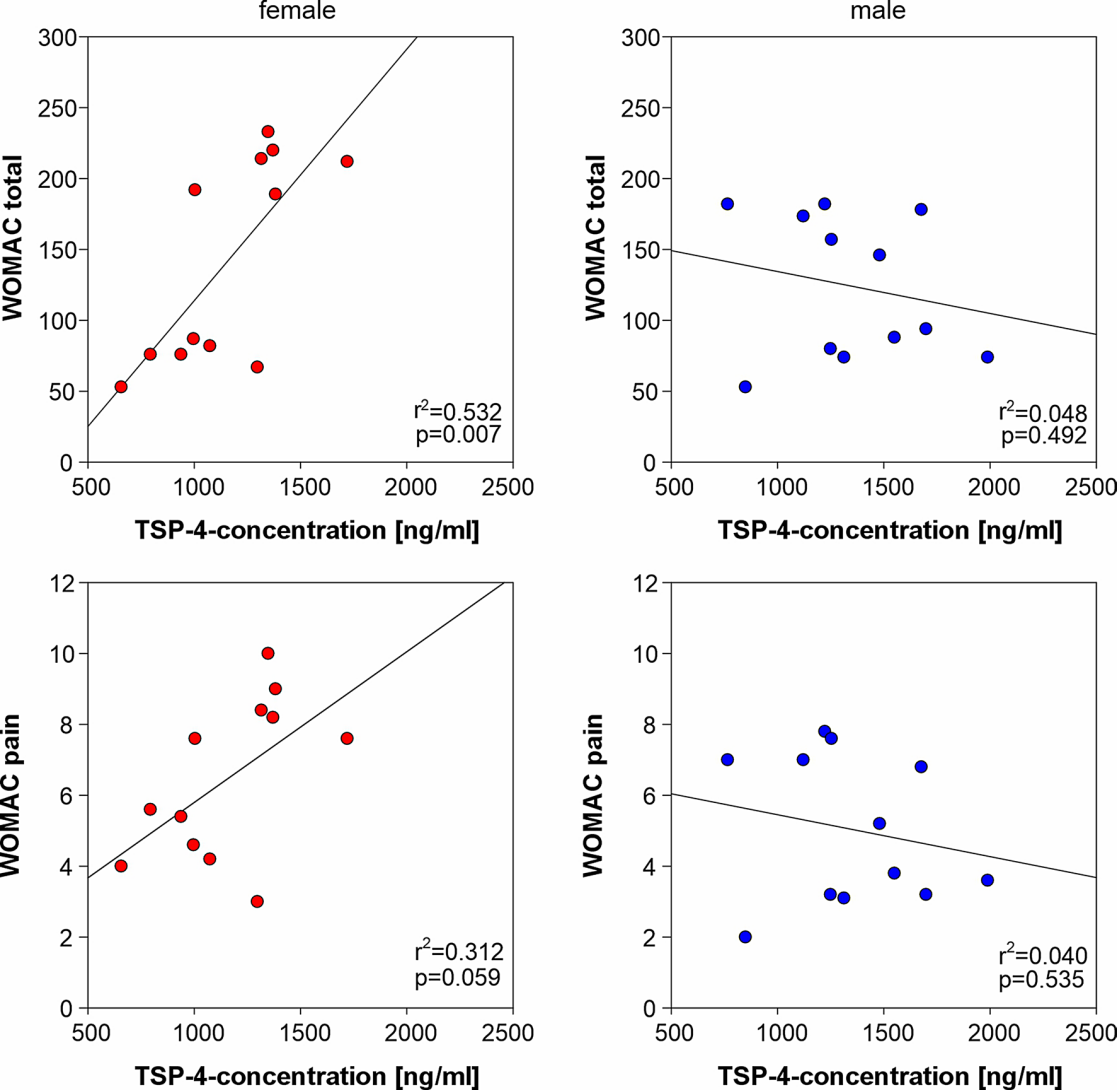
Association of synovial fluid TSP-4 concentration with WOMAC scores. Correlation between synovial fluid TSP-4 concentration and total WOMAC and WOMAC pain scores in female (red circles, *n* = 12) and male (blue circles, *n* = 12) OA patients (total *n* = 24). r^2^ values indicate the coefficient of determination and p values indicate the level of significance

### Correlation between TSP-4 expression in IFP and synovial fluid

**Fig. 7:**
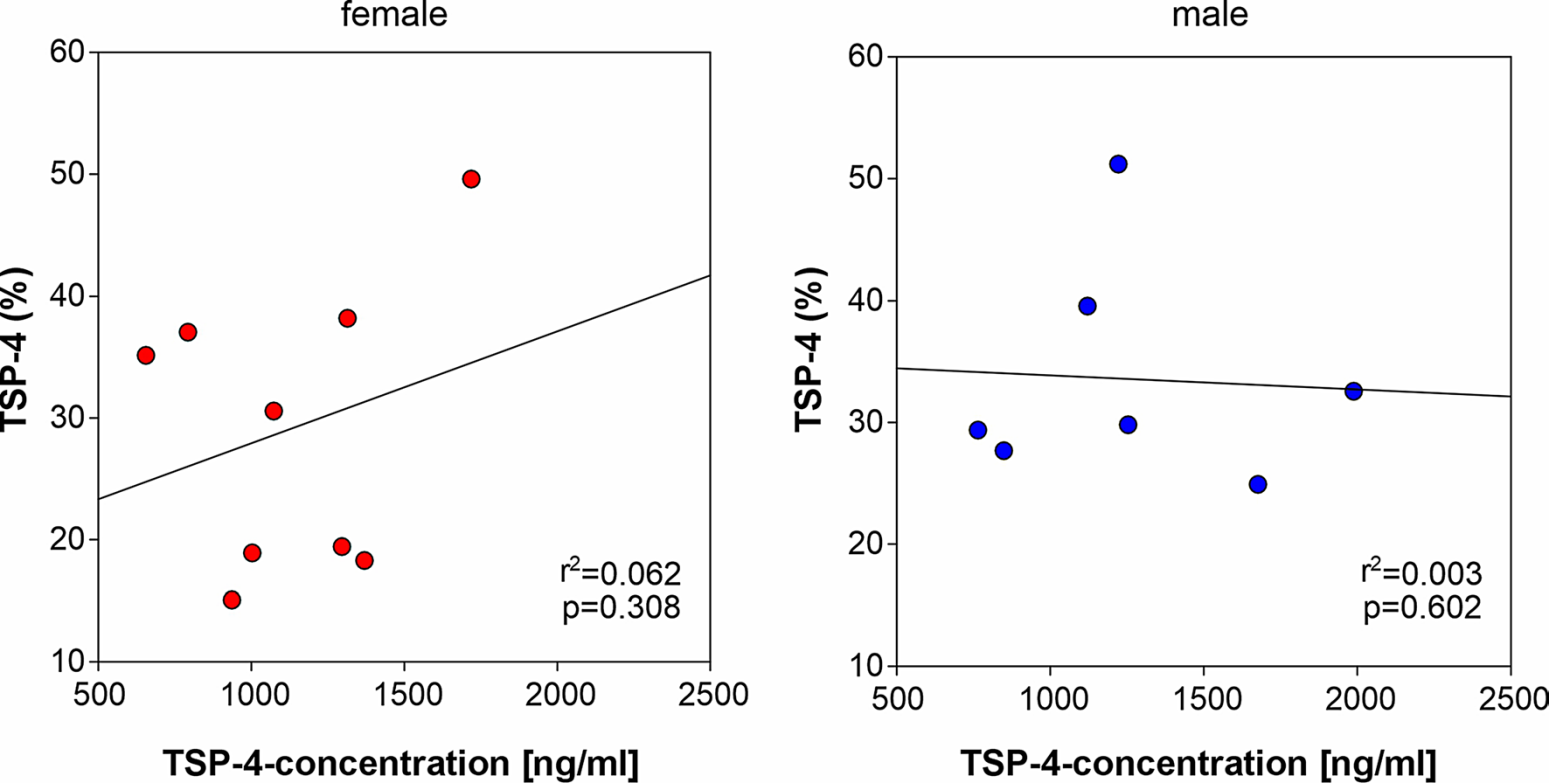
Correlation between TSP-4 in IFP and synovial fluid. Relationship between TSP-4-positive area in IFP tissue and TSP-4 concentration in the synovial fluid of female and male patients. Each red circles represent individual female patients (*n* = 9) and blue circles represent individual male patients (*n* = 7). r^2^ indicates the coefficient of determination and p value indicates the level of significance

To elucidate, whether the IFP contributes significantly to TSP-4 levels detected in synovial fluid, the percentage of TSP-4-positive area in IFP section was correlated with synovial fluid TSP-4 concentration. However, there was no significant correlation between these parameters (Fig. 6, 7).

## Discussion

OA remains the most prevalent chronic degenerative joint disease resulting in pain [27]. Despite advances, the roles of various joint tissues in OA pathogenesis, particularly the contribution of the IFP is not fully understood. It is well known that the IFP secretes many factors that might drive the disease progression [2]. In addition, fibrotic changes were observed in the IFP. In a recent study, an increased expression of the extracellular matrix protein COMP correlated with the grade of fibrosis in the IFP [14]. However, whether other members of the thrombospondin family that interact with collagens and are related to fibrotic processes are also expressed has not yet been investigated. Interestingly, TSP-4 has been shown to be upregulated in several disease models associated with pain [28]. Consequently, this study aimed to elucidate, whether TSP-4 is expressed in the IFP of patients with late stage OA and whether its expression correlates with fibrotic changes and/or knee joint pain.

Our study demonstrated a clear correlation between degree of fibrosis, vascularization and lymphocytic infiltration in IFP of OA patients undergoing TKA, illustrating that a highly fibrotic IFP is not only well vascularized but also exhibits inflammatory changes. This connection appears to be consistent and independent of variables such as BMI, age, or sex. These results are consistent with previously published data [29-31].

We here demonstrated for the first time that TSP-4 is expressed in the IFP predominantly in fibrotic areas. In general, TSP-4 expression is relatively low in healthy connective tissues but increases during degenerative and remodeling processes. In articular cartilage, its expression strongly increases with the onset of OA and persists in later stages [16]. Besides that, increased levels of TSP-4 were detected in bone marrow lesions of OA patients [18] and TSP-4 has also been identified in transient cartilage during ossification and repair processes, where it may be related to neovascularization [32]. In other tissues such as the myocardium or skin, TSP-4 was identified as a strong regulator of fibrosis [33], however, its pro- or anti-fibrotic properties are tissue-specific [34]. In the IFP of OA patients, we detected a strong positive correlation between the area of fibrosis and TSP-4 expression indicating a more pro-fibrotic role. Moreover, in our patient cohort, we identified a subgroup with low fibrosis but increased TSP-4 levels. This might suggest that an increase in TSP-4 expression initiates fibrotic tissue remodeling as also demonstrated in hypertrophic scar formation [33]. As we demonstrated in an earlier study, TSP-4 interacts directly with TGF-β [35]. TGF-β is a strong inducer of fibrotic processes and it is likely that the TSP-4/TGF-β complex enhances the fibrotic response as already described for the COMP/ TGF-β complex [36].

Besides its role in connective tissue remodeling, an upregulation of TSP-4 has been also associated with genesis of pain in different animal pain models [19, 21, 23]. Previous studies demonstrated that induced peripheral nerve injury or spinal cord contusion trigger an upregulation of TSP-4 in the dorsal spinal cord, which seems to be crucial for the development of neuropathic pain. Conversely, genetic blocking or inactivation of increased expression of TSP-4 also leads to reduced hypersensitivity, and induced neuropathic pain states can be reversed [19, 20]. In a study using an orofacial pain model, this relationship was also confirmed [21]. In another study by Crosby et al., the neuronal interactions of TSP-4 were also demonstrated in joint-mediated pain [22, 23]. In addition, TSP-4 has recently been shown to impact sensory afferent terminals in the spinal cord to stimulate excitatory synaptogenesis and contribute to the phenomenon of central sensitization [20, 24], suggesting that TSP-4 is an important contributor to neuropathic pain which is commonly observed in late OA. There was no significant correlation between TSP-4 expression level in IFP tissue and neither total WOMAC nor WOMAC pain scores. In two patients, high levels of fibrosis and TSP-4 were associated with high total WOMAC and WOMAC pain scores. The fact that we never observed low WOMAC scores in combination with high fibrosis or high TSP-4 levels might suggest a correlation between these parameters and pain. However, this has to be investigated in larger cohorts in future studies. It has been previously demonstrated that TSP family proteins, in particular TSP-4, promote synaptogenesis [37]. Furthermore, TSP-4 directly interacts with the calcium channel alpha2delta1 subunit resulting in excitatory synapse formation in sensory neurons and consequently, in increased pain levels [38]. This interaction is antagonized by gabapentin that is in some cases used to relieve severe pain in knee OA [37].

The fact that no significant correlation between TSP-4 expression level in IFP tissue and pain was found in our study could also be explained by a release of TSP-4 from other joint tissues accumulating in the synovial fluid. Indeed, we were able to detect TSP-4 levels in a similar order of magnitude as previously demonstrated for COMP [39]. There was no significant correlation between synovial TSP-4 concentrations WOMAC scores in general. However, when considering potential sex-specific differences, the data analysis revealed a significant and strong correlation in female but not in male patients. Cui et al. identified a genotype variant of TSP-4 that is a risk factor for the development of myocardial infarction only in women [55,56]. However, the reason for this sex-dependent difference remains unknown and has to be addressed in future studies. Interestingly, TSP-2 showed a sex-specific difference in synaptogenesis with a more robust synaptogenic response in male rats [40]. This is in contrast to our results on TSP-4 and pain, however, it has been reported that although they share a similar molecular structure, different members of the TSP family can exert opposing effects [41]. To date, there is no study published on sex-specific differences regarding TSP-4 levels in synovial fluid but genetic, hormonal, psychosocial factors, or the disease progression itself may have an influence [42].

TSP-4 levels in IFP and synovial fluid did not correlate and there are at least two reasons for this observation. First, other joint tissues than IFP might contribute to the synthesis and accumulation of TSP-4 in the synovial fluid, for example cartilage or synovial tissue [16]. Second, TSP-4 might be rapidly degraded by synovial matrix metalloproteases that are upregulated by OA [43]. Tissue-specific proteolytic cleavage of ECM components might also give rise to bioactive fragments driving OA pain as shown previously for an aggrecan fragment [44].

Certainly, our study has some limitations. It was initiated as a pilot study and therefore, the study population is relatively small and our original findings should be validated in larger cohorts in the future. Another potential limitation is the heterogeneity within IFP samples from an individual patient. For this reason, we evaluated 25 areas per patient and calculated mean values to provide a reliable measure of the overall changes within their IFPs. Moreover, analgesic drugs are quite often used among OA patients and might influence WOMAC scores. In addition, WOMAC scores are subjective measures and their correlation with further parameters might not always reflect the pain levels without medication.

## Conclusion

For the first time, TSP-4 expression was demonstrated in the IFP. The correlation between TSP-4 staining intensity and degree of fibrosis and in particular the fact that increased TSP-4 intensity was observed in sections with minor fibrotic changes suggests that TSP-4 might play a role in the development of IFP fibrosis. While TSP-4 in IFP does not seem to contribute to genesis of pain, TSP-4 in synovial fluid might play an important role in this regard. The analysis of synovial fluid and serum samples in larger patient cohorts will show if TSP-4 could be used as a pain biomarker or even represent a novel analgesic target.

## Electronic supplementary material

Below is the link to the electronic supplementary material.

Supplementary Material 1

## Data Availability

The data analyzed during the current study are available from the corresponding author on reasonable request.

## Abbreviations

BMI: Body mass index
COMP: Cartilage oligomeric matrix protein
DAB: 3,3’-Diaminobenzidine
ELISA: Enzyme-linked immunosorbent assay
HE: Hematoxylin-eosin
HRP: Horseradish peroxidase
IFP: Infrapatellar fat pad
IgG: Immunoglobulin G
NOVA: Analysis of variance
NSAID: Non-steroidal anti-inflammatory drugs
OA: Osteoarthritis
PBS: Phosphate-buffered saline
SD: Standard deviation
TGF-β: Transforming growth factor β
TKA: Total knee arthroplasty
TSP-4: Thrombospondin-4
WOMAC: Western Ontario and McMaster universities osteoarthritis index
