# Supplementary material for: Expression of thrombospondin-4 in the infrapatellar fat pad and synovial fluid – potential contribution to osteoarthritis pain
Source: Arthritis Res Ther. 2025 Aug 26;27:170. doi: 10.1186/s13075-025-03615-7 (PMC12379533; doi:10.1186/s13075-025-03615-7)
Supplement: Supplementary file 1 — Supplementary Material 1 [file 13075_2025_3615_MOESM1_ESM.docx]

**Supplementary Material**

**Expression of Thrombospondin-4 in the Infrapatellar Fat Pad and Synovial Fluid – Potential Contribution to Osteoarthritis Pain**

Sebastian Braun^2^, Patrizia Pollinger^1^, Rebecca Sohn^1^, Anna E. Rapp^1^, Gundula Rösch^1^, Frank Zaucke^1^, Zsuzsa Jenei-Lanzl^1*^

^1^ Dr. Rolf M. Schwiete Research Unit for Osteoarthritis, Department of Trauma Surgery and Orthopedics, Goethe University Frankfurt, University Hospital, Marienburgstraße 2, 60528 Frankfurt am Main, Germany

^2^ Charité – Universitätsmedizin Berlin, corporate member of Freie Universität Berlin and Humboldt-Universität zu Berlin, Center for musculoskeletal Surgery, Charitéplatz 1, 10117 Berlin, Germany


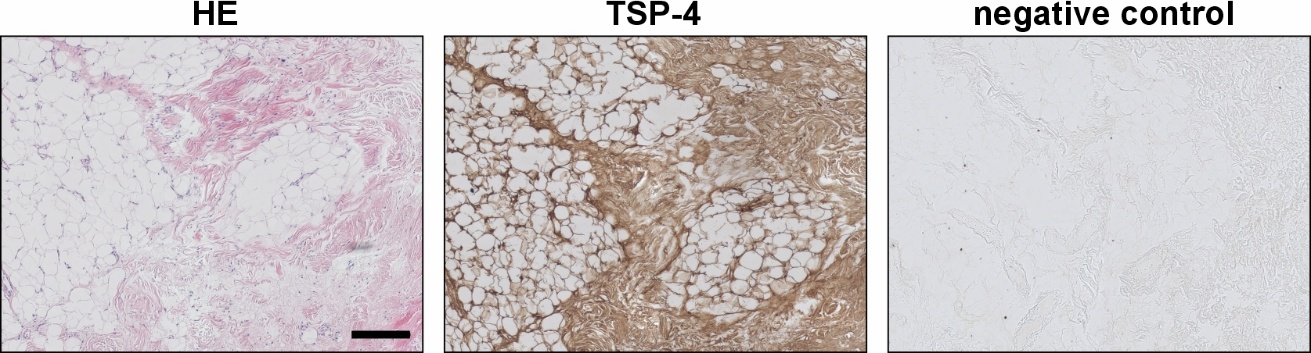


**Supplementary Figure 1:** HE staining and TSP-4 immunohistochemistry of a representative section with medium fibrosis and the corresponding negative control to the TSP-4 staining (no primary antibody, scale bar 200 µm).
